# Role of CD8^+^ T cells in protection against *Leishmania donovani* infection in healed Visceral Leishmaniasis individuals

**DOI:** 10.1186/s12879-014-0653-6

**Published:** 2014-12-03

**Authors:** Himanshu Kaushal, Rachel Bras-Gonçalves, Narender Singh Negi, Jean-Loup Lemesre, Gérard Papierok, Poonam Salotra

**Affiliations:** National Institute of Pathology (ICMR), Safdarjung Hospital Campus, New Delhi, 110029 India; Institut de Recherche pour le Développement, UMR177 IRD/CIRAD “INTERTRYP”, Montpellier, France; Department of Medicine, VMMC & Safdarjung Hospital, New Delhi, India; Virbac, Carros, France

**Keywords:** CD8+ T cells, Granzyme B, Visceral leishmaniasis, Total soluble Leishmania antigens, Vaccine

## Abstract

**Background:**

Majority of individuals with history of visceral leishmaniasis (VL) exhibit strong immunity to re-infection, however, the mechanism of resistance is poorly understood. It is unclear whether CD8^+^ T cells contribute to protection against *Leishmania donovani* infection through cytotoxic activity. The present study aims to evaluate immunological mechanism associated with resistance to the disease in healed VL (HVL) individuals and further, the contribution of CD8^+^ T cells in the protective immunity.

**Methods:**

Peripheral blood mononuclear cells (PBMCs) from VL, HVL and naive groups were exposed *in vitro* to total soluble *Leishmania* antigen (TSLA) from *L. donovani*. The proliferation index was determined by ELISA based lymphoproliferative assay. Cytokines and granzyme B levels were measured by CBA. Activated T-cell populations were estimated using flow cytometry.

**Results:**

We observed significantly higher lymphoproliferation, cytokines and granzyme B levels in HVL group compared to naive or VL group. More strikingly, we found a strong association (r_s_ = 0.895, *P* < 0.0001) between proliferation index (PI) and granzyme B level, with a significant proportion of activated CD8^+^ T cells in HVL group.

**Conclusions:**

*Leishmania* immune group (HVL) exhibited durable and strong cellular immune response to TSLA in terms of lymphoproliferation as well as production of Th1 cytokines and granzyme B. Additionally, the elevated level of activated CD8^+^ T cells and stimulation of cytotoxic activity through granzyme B production, indicated a possible role of CD8^+^ T cells in resistance to *L. donovani* infection in the HVL group.

**Electronic supplementary material:**

The online version of this article (doi:10.1186/s12879-014-0653-6) contains supplementary material, which is available to authorized users.

## Background

Leishmaniasis is a neglected tropical disease which poses risk to over 350 million people worldwide, in 98 countries or territories [1]. Depending on the species, the disease manifests into different clinical forms, ranging from self-healing cutaneous leishmaniasis (CL) to disfiguring mucosal lesions to the visceral form, visceral leishmaniasis (VL). Of these, VL is the most severe form which proves fatal if diagnosed late or left untreated. Currently, more than 90% of annual incidence of VL occurs in countries like India, Sudan, Bangladesh, Brazil, Ethiopia and the Nepal [2],[3]. In India, about 5–10% of apparently healed VL patients develop an unusual dermal form of the disease termed Post-kala azar dermal leishmaniasis (PKDL) which constitutes an important reservoir for the parasites [4].

Previous studies have shown that majority of individuals who had VL or asymptomatic infection acquired strong immunity against re-infection with the same subspecies [5],[6]. This observation strongly advocates the development of an anti-leishmanial vaccine which could induce a long lasting immunity similar to that acquired naturally in healed visceral leishmaniasis (HVL) individuals. However, understanding the immunological mechanism associated with resistance and susceptibility to disease is of utmost importance for the design and evaluation of a vaccine. This aspect is understudied in human VL although several reports are available in murine models for both CL and VL.

Immunopathology of human VL presents a mixed T-helper 1 (Th1)/T-helper 2 (Th2) cytokine response, and is characterized by the presence of a dominant Th2 response over Th1 response. However, this response gets reversed in HVL individuals with up regulation of Th1 response [7],[8]. The T lymphocyte profile of peripheral blood mononuclear cells (PBMCs) of active VL patients have higher proportion of CD8^+^ T cells compared to CD4^+^ T cells which approach normal levels post-treatment [9],[10]. Furthermore, it was recently revealed that there is complete anergic/exhaustion in CD8^+^ T cells in chronic VL patients, with limited ability to contribute IFN-γ [11].

In experimental models, the role of CD8^+^ T cells in the control of murine CL and determination of resistance to re-infection remains contradictory. However, CD8^+^ T cells have been thought to play a major role in murine VL. CD8^+^ T cells participate not only in primary but also in subsequent infection with *Leishmania donovani* [12]. One study revealed that purified CD8^+^ T cells from the mice infected with *L. infantum* expressed Th1 cytokines (IFN-γ and TNF-α), and showed considerable cytotoxic activity [13]. Another study in murine model suggested that *L. donovani* escapes cellular responses by inducing exhaustion in CD8^+^ T cells [14], however, in canine VL, both CD4^+^ and CD8^+^ T cells show Programmed Death-1 mediated exhaustion, which impairs their phagocyte function [15].

In human leishmaniasis, the role of CD8^+^ T cells is not clear and depends largely on the species and the corresponding disease. Limited studies have been carried out with VL patients and ascribe a protective role for CD8^+^ T cells, which is similar as in mice models. One earlier study showed involvement of not only IFN-γ producing CD4^+^ T cells, but also CD8^+^ T cells in the control of *L. infantum* infection [16]. In human CL, the exact role of CD8^+^ T cells is unclear. In a recent study, a major correlation was observed between protection and the CD8^+^ T cells producing IFN-γ after re-stimulation [17]. In humans, several studies suggest that CD8^+^ T cells mediate protection through cytotoxicity against intracellular pathogens, such as *Trypanosoma cruzi* and *Mycobacterium tuberculosis* [18]-[20]. However, it is unclear whether CD8^+^ T cells contribute protection against *L. donovani* parasites through the cytotoxic activity.

In the present study, *Leishmania*-specific cellular immune responses upon *in vitro* stimulation of PBMCs with total soluble *Leishmania* antigen (TSLA) were evaluated in VL, HVL and naive groups by measuring lymphoproliferation, cytokines, granzyme B and the proportion of activated T cell populations. The data suggested the possible role of effector CD8^+^ T cells in resistance to *L. donovani* infection in HVL individuals.

## Methods

### Study subjects

Our study included active VL (n = 11), HVL (n = 16) and naive (n = 19) individuals, all seronegative for HIV and above sixteen years of age. Patients clinically diagnosed with VL (age range in years, 17–62; age mean ± SD, 36.54 ± 14.37), were admitted to Department of Medicine, Safdarjung Hospital, New Delhi. Active VL was diagnosed with the clinical features such as fever, hepatosplenomegaly, anaemia, weight loss, and pancytopenia and positive rK39 strip test. VL was confirmed by presence of Leishman-Donovan (LD) bodies and/or by PCR in bone marrow aspirates. Individuals included in HVL group (age range in years, 19–48; age mean ± SD 31.4 ± 9.19) were healthy individuals that had completed VL treatment at least one year back. The range of post-treatment duration for the HVL group was 1 to 20 yrs, with mean ± SD 11 ± 5.76 in yrs. They were all positive for rK39 strip test and majority (15/16) were PCR negative. Blood samples of 19 naive individuals (age range, 18–35; age mean ± SD 26.89 ± 4.56) were included in this study. All naive individuals were negative for rK39 strip test and for lymphoproliferative assay.

### Ethics statement

The study was approved by and carried out under the guidelines of the ethical committee of the Safdarjung Hospital, India. All individuals under study provided written informed consent for the collection of samples and subsequent analysis.

### Preparation of Total Soluble *Leishmania*antigen (TSLA)

Promastigotes of *L. donovani* (MHOM/IN/80/Ldd8Cl2) were harvested in stationary phase, washed and the pellet resuspended in the lysing solution (50 mM Tris/5 mM EDTA/HCl, pH7). After three cycles of freezing/thawing, the samples were subjected to three pulses of 20 seconds at 40 W with sonicator, at one minute interval. The sample was centrifuged at 5000 × g for 20 min at 4°C, and supernatant was collected. Protein content was estimated using Bradford method. TSLA was aliquoted and stored at −80°C until further use.

### Lymphoproliferative assay

PBMCs were isolated from the heparinised blood samples by density sedimentation (Ficoll-PaqueTM PLUS; GE Healthcare), washed, resuspended in RPMI 1640 supplemented with 10% FCS, penicillin (100 U/ml), and streptomycin (100 μg/ml). PBMCs at concentration of 1 × 10^6^ cells/ml were cultured in triplicate in 96-well flat-bottom tissue culture plates (Axygen, Union city, CA, USA) and stimulated with TSLA (10 μg/ml) or PHA-M (10 μg/ml) for 120 hrs in humidified 37°C/5% CO_2_ incubator. At 104–106 hrs incubation, 20 μL BrdU labelling solution was added and samples reincubated in humidified 37°C/5% CO_2_ incubator for another 16–18 hrs. Lymphoproliferation was evaluated by commercially available kit (BiotrakTM cell proliferation ELISA system, version 2, GE Healthcare) using ELISA method. The proliferation index (PI) was calculated as the ratio of optical density (OD) of stimulated cultures and unstimulated cultures for each sample. Cell proliferation was considered significant when PI was above cut-off (mean + 3SD).

### Estimation of cytokines and granzyme B in culture supernatant

PBMCs of active VL, HVL and naive individuals were incubated with TSLA for 120 hrs as described above. The tissue culture plates were centrifuged and supernatants were collected and stored at −80°C until use. Cytokines (IFN-γ, TNF-α and IL-10) and granzyme B levels were analysed by utilising cytometric bead array (CBA) flex sets (BD Biosciences) and measuring fluorescence by flow cytometry according to manufacturer’s recommendations. Samples were acquired on flow cytometer, BD FACSCalibur using BD CellQuest Pro software and the data were analysed using FCAP array software (BD Biosciences). The assay sensitivity for IFN-γ, TNF-α, IL-10 and granzyme B were 1.8, 1.2, 0.13 and 4 pg/ml respectively.

### Flow cytometer analysis for cell surface phenotype of activated T cell populations

Detection of activation in CD4^+^ or CD8^+^ T cells were done by freshly isolated PBMCs (10^6^/ml) from eight HVL and eight naive individuals within recruited individuals for this study, and incubated with TSLA (10 μg/ml) in a 96-well flat-bottom plate for 120 hrs at 37°C. After 120 hrs, the cells were harvested, washed with staining buffer (0.02 M PBS, 1% FBS, and 0.01% sodium azide), and surface stained with fluorochrome-conjugated antibodies to CD3-FITC, CD4-PerCP-Cy5.5, CD8-PerCP-Cy5.5 and CD69-APC, along with appropriate isotype controls (BD Biosciences) for 30 min at 4°C. Cells were then washed and finally suspended in 500 μl staining buffer (BD Biosciences). Samples were acquired and analyzed on flow cytometer, BD FACSCalibur using BD CellQuest Pro software on at least 10,000 events. Analysis gates were set for lymphocytes using forward and side scatter properties and the frequencies of activated CD4^+^ and CD8^+^ T cells were acquired onCD3^+^ T cells. Cell viability using 7AAD staining (BD) of a limited number of samples confirmed that the gated lymphocytes were >99% viable for both HVL and naive groups.

### Statistical analysis

Results are represented as mean ± SE. Data were analysed using GraphPad Prism 5.0 (GraphPad Software, San Diego, CA). Statistical significance were determined by nonparametric Mann–Whitney test between two groups and by Kruskal-Wallis test followed by the post hoc Dunn multiple comparison test for more than two groups. Correlation was calculated by Spearman rank correlation test. The statistical tests were two-tailed and *p* values < 0.05 were considered significant.

## Results

### Proliferative response of peripheral lymphocytes to *L. donovani*TSLA

**Figure 1:**
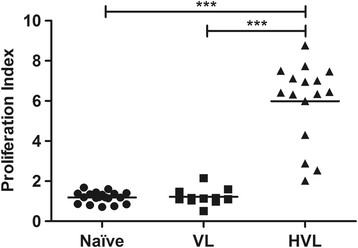
**Lymphoproliferative response to TSLA in VL, HVL and naive groups.** Peripheral blood lymphocytes from individuals with active VL (n = 11), HVL (n = 16) and naive (n = 19) groups were incubated with TSLA (10 μg/ml) for 120 hrs and lymphoproliferation was measured by BrdU incorporation for the last 12–14 hrs using BiotrakTM cell proliferation ELISA system. Horizontal lines indicate mean values. ***p < 0.001.

Cell-Mediated Immune (CMI) response was analyzed in terms of lymphoproliferative response *in vitro* to TSLA and phytohemagglutinin (PHA). PHA served as positive control and every individual of the three study group showed high stimulation (Naive PI mean ± SE, 10.30 ± 3.649; VL, 7.73 ± 3.893; HVL, 11.30 ± 3.03). Every individual in HVL group responded positively to TSLA and the group mean (PI mean ± SE, 5.99 ± 0.497), was found significantly high (*p* < 0.001) compared to naive group (PI mean ± SE, 1.19 ± 0.064) or active VL (PI mean ± SE, 1.22 ± 0.124) (Figure 1). The response in active VL was found comparable to naive group (*p* = 0.546).

### Estimation of cytokines upon TSLA stimulation

**Figure 2:**
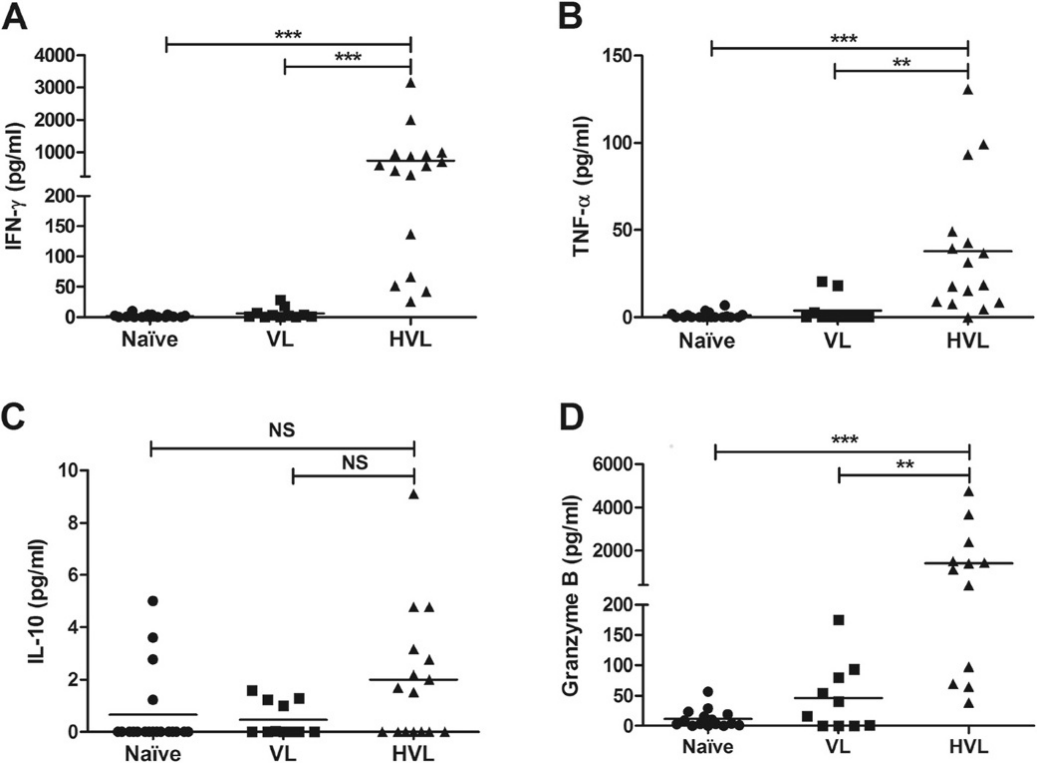
***In vitro Leishmania*** **-specific cellular immune response in VL, HVL and naive groups.** PBMCs were isolated and incubated with TSLA (10 μg/ml) for 120 hrs. **(A-C)** Cytokines (IFN-γ, TNF-α and IL-10) were measured in the supernatant of VL (n = 11), HVL (n = 16) and naive (n = 19) and **(D)** Granzyme B were analyzed in the supernatant of VL (n = 11), HVL (n = 12) and naive (n = 16) using CBA. Horizontal lines indicate mean values. NS, Not Significant, **p < 0.01, ***p < 0.001.

PBMCs from active VL, HVL and naive groups were screened for cytokines profile in response to TSLA (Figure 2A-C). In HVL group, the CMI response (as judged from the secretion of Th1 cytokines, IFN-γ and TNF-α) was found significantly higher than the naive or active VL. HVL group (mean ± SE, 738.44 ± 206.21) showed significantly high (*p* < 0.001) IFN-γ level compared to naive (mean ± SE, 1.80 ± 0.57) or active VL (mean ± SE, 10.63 ± 2.76). Cut off (Mean + 3SD) value for IFN-γ was determined as 9.33 pg/ml and, importantly, every HVL individual showed IFN-γ level above cut-off value. Similarly, significantly high TNF-α production was observed in response to TSLA stimulation in HVL group (mean ± SE, 37.71 ± 9.59) compared to naive (mean ± SE, 1.01 ± 0.43, *p* < 0.001) or active VL (mean ± SE, 3.77 ± 2.33, *p* < 0.01). For IL-10 cytokine, the measured values for HVL (mean ± SE, 2 ± 0.63) were low, and comparable to naive, (mean ± SE, 0.66 ± 0.33) or active VL (mean ± SE, 0.69 ± 0.19).

### Granzyme B analysis

Granzyme B, a serine proteinase produced by the cytotoxic lymphocytes, notably, induces rapid cellular death of the target by apoptosis. Here, granzyme B was measured in culture supernatant upon *in vitro* TSLA stimulation of PBMCs. The granzyme B level of HVL group (mean ± SE, 1411.91 ± 441.62), was found significantly high compared to naive (mean ± SE, 11.79 ± 3.98, *p* < 0.001) or active VL group (mean ± SE, 45.97 ± 18.08, *p* < 0.01), with 11/12 (92%) HVL individuals showing values above cut off value (58.10 pg/ml) (Figure 2D). Mean of active VL group was found comparable to naive (*p* = 0.371).

### Correlation between granzyme B level and proliferation index

**Figure 3:**
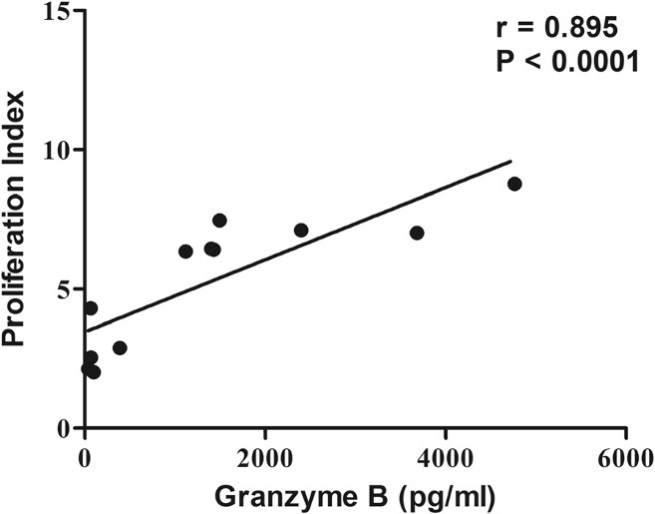
**Comparative assessment of granzyme B level and proliferation index upon TSLA stimulation of PBMCs in HVL (n = 12) group.** Correlation was calculated by Spearman rank correlation test. Diagonal line represents the best fit line.

The secretion of granzyme B upon TSLA stimulation were analysed in active VL, HVL and naive group with the respective proliferation index. In HVL group, the level of granzyme B was found strongly correlated with PI (r_s_ = 0.895, *p* < 0.0001) (Figure 3). Besides, we observed moderately significant correlation between PI and IFN-γ and TNF-α with r_s_ = 0.63 (*p* = 0.03) and r_s_ = 0.62 (*p* = 0.03) respectively in HVL group. However, we did not observe any significant correlation between granzyme B and IFN-γ level (r_s_ = 0.434, *p* = 0.158) in the culture supernatant.

### Estimation of activated T lymphocytes

**Figure 4:**
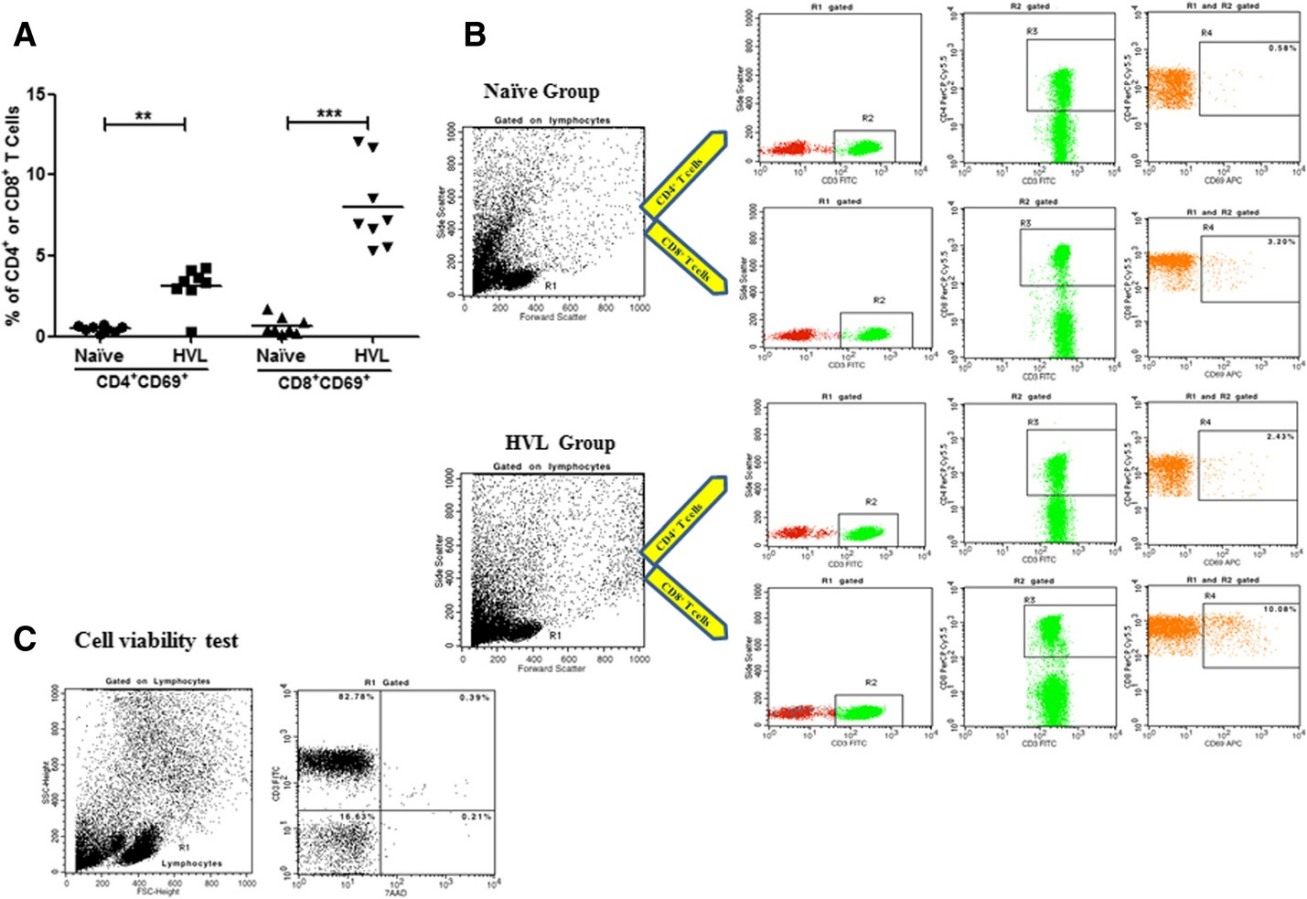
**Estimation of activated T lymphocytes. (A)** Percentage of TSLA-activated CD4^+^ and CD8^+^ T cells in HVL and naive groups. PBMCs from HVL (n = 8) and naive (n = 8) were incubated with TSLA (10 μg/ml) for 120 hrs at 37°C. The values of unstimulated cells were substracted from TSLA stimulated cells. **(B)** Data showing representative FACS analysis in one each from HVL and naive individuals. Analysis gates were set for lymphocytes using forward and side scatter properties and the frequencies of activated CD4^+^ and CD8^+^cells were acquired on CD3^+^ T cells. **(C)** Cell viability test using 7AAD staining was done and the data show a representative FACS analysis in one of HVL individuals. Horizontal lines indicate mean values. *p < 0.05, **p < 0.01.

The strong association between PI and granzyme B in the HVL group, provided lead for the possible protective role of CD8^+^ T cells. Therefore, using CD69 as a marker of activation [21], we investigated the percentage of activated CD4^+^ and CD8^+^ T cell populations upon *in vitro* TSLA stimulation both in HVL and naive groups. CD8^+^ T cells showed a pronounced activation in HVL, with significantly higher percentage of CD8^+^CD69^+^ T cell population in HVL group (mean ± SE, 7.99 ± 0.91, *p* = 0.0002) compared to naive group (mean ± SE, 0.67 ± 0.19). There was also a significantly higher percentage of CD4^+^CD69^+^ T cell (mean ± SE, 3.12 ± 0.44, *p* = 0.0047) in HVL compared to naive group (mean ± SE 0.46 ± 0.07) (Figure 4).

## Discussion

In the present study, we initially evaluated CMI response in terms of lymphoproliferation upon *in vitro* TSLA stimulation in active VL, HVL and naive groups. We demonstrated significantly high lymphoproliferation in *Leishmania* immune group (HVL) implying the presence of circulating *Leishmania*-specific memory T cells which showed significantly higher lymphoproliferation compared to that in unexposed individuals (naive). On the contrary, active VL group failed to show lymphoproliferation which indicated immune dysfunction [11],[22]. Besides, earlier studies investigated cellular immune responses in HVL individuals with short VL history (up to 1 year) [11],[23],[24] whereas the present study included individuals with long history of VL (1 to 20 yrs, mean ± SD, 11 ± 5.76 yrs) with all cases showing PI values well above cut-off, indicating that an anti-leishmanial vaccine could provide long term protection to *Leishmania* infection.

The cytokine analysis constitutes an important part since they form a complex network of synergistic and antagonistic interactions which not only induce but also control immune response. IFN-γ, produced predominantly by activated CD4 Th1 and CD8 cytotoxic T lymphocyte (CTL) and NK cells in response to IL-12 signaling, is an important activator of macrophages that enhances their microbicidal activity against intracellular pathogens [25],[26]. It promotes NO production by inducing iNOS (inducible nitric oxide synthase) expression by infected phagocytes thus facilitating elimination of parasites and resolving *Leishmania* infection [27]. Another Th1 cytokine, tumor necrosis factor alpha (TNF-α), known to exert cytotoxic effects on pathogens, has been closely associated with VL pathogenesis, being low in active VL and getting restored after treatment [28]. The cytokine profile upon TSLA stimulation of PBMCs corroborates our lymphoproliferation data. The production of significantly higher level of Th1 cytokines (IFN-γ and TNF-α) to TSLA was observed in HVL group compared to naive or active VL group and is in accordance with earlier studies [29]-[31]. The significant association between PI and IFN-γ and TNF-α in HVL group indicates the strong CMI in individuals with prior exposure to *Leishmania* antigens. As expected, no protective immune response was observed in naive individuals while the active VL group exhibited peripheral lymphocytes anergy [22]. IL-10 on the other hand has counter regulatory role against IL-12 and IFN-γ and thus favours the survival of *Leishmania* parasites by inhibiting NO-mediated killing [32]. We observed no significant difference in IL-10 production between the different study groups.

There are mainly two mechanisms by which cytotoxic cells lyse their targets: the perforin-granzyme B pathway and death receptors (Fas/FasL) [33],[34]. The FasL-dependent pathway utilises Fas surface receptor by Fas ligand expressed on the surface of the CTL and NK Cells, which triggers Fas-mediated apoptosis in target cells. The chief mechanism used by cytotoxic cells to induce target cell death is through the granule exocytosis pathway and depends on the concerted action of effector molecules contained in the cytolytic granules. These granules contain perforin, the pore-forming molecule, together with granule-associated enzymes. Among them, granzyme B is the most important effector molecule for target-cell apoptosis [35]. It is unclear whether CD8^+^ T cells contribute protection against *L. donovani* parasites through their cytotoxic activity. Limited studies have been conducted dealing with parasite-specific cell-mediated cytotoxicity in VL, CL or mucocutaneous leishmaniasis [31],[36]-[38]. Here, we evaluated granzyme B level to investigate whether individuals healed after *L. donovani* infection develops a cytotoxic immune response upon re-exposure. We demonstrated significantly higher granzyme B level upon TSLA stimulation in *Leishmania* immune (HVL) group compared to naive or VL group, with a strong association between PI and granzyme B level. Previous studies with viral infections have shown that granzyme B is predominantly secreted by CD8^+^ T cells [39], although, the contributions of NK cells and CD4^+^ cytotoxic cells have also been suggested [40],[41]. Furthermore, we observed a significantly high percentage of CD8^+^CD69^+^ and CD4^+^CD69^+^ T cells in the healed VL individuals. This is distinct from the report in cured CL individuals where a high CD4^+^CD69^+^ T cells was demonstrated to be responsible for the immunity to *L. major* infection [31].

## Conclusion

The study brings forth some essential points regarding the immunological mechanism associated with resistance to VL in healed VL individuals with long history of VL. The preponderance of CD8^+^ T cells was suggested in resistance to *L. donovani* infection possibly via the perforin-granzyme B pathway and by the activation of significant proportion of CD8^+^ T cell populations. The findings support the role of CD8^+^ T cells in resistance to *Leishmania* infection, which could be exploited for the design and evaluation of a vaccine.

## Authors’ original submitted files for images

Below are the links to the authors’ original submitted files for images. Authors’ original file for figure 1 Authors’ original file for figure 2 Authors’ original file for figure 3 Authors’ original file for figure 4

## Abbreviations

VL: Visceral leishmaniasis
PKDL: Post kala-azar dermal Leishmaniasis
CL: Cutaneous leishmaniasis
IL: Interleukin
IFN: Interferon
Th: T Helper cell
TNF: Tumour necrosis factor
TSLA: Total soluble *Leishmania* antigen
PBMCs: Peripheral blood mononuclear cells
