# Supplementary figures and images for: Role of CD8+ T cells in protection against Leishmania donovani infection in healed Visceral Leishmaniasis individuals
Source: BMC Infect Dis. 2014 Dec 3;14:653. doi: 10.1186/s12879-014-0653-6 (PMC4258298; doi:10.1186/s12879-014-0653-6)

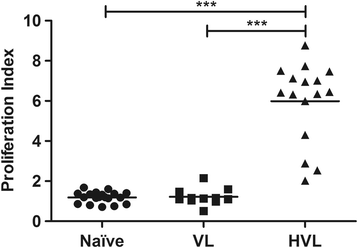

Supplement: Supplementary file 1 — Authors’ original file for figure 1 [file 12879_2014_653_MOESM1_ESM.gif]

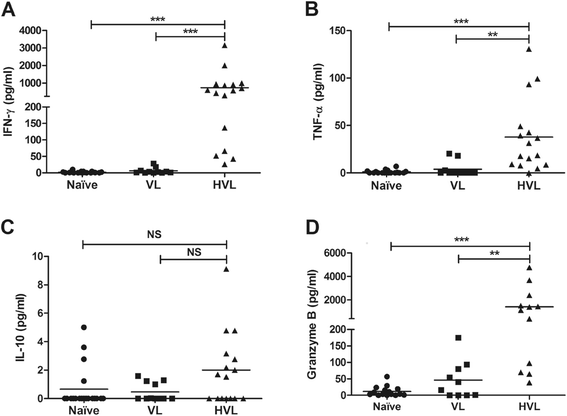

Supplement: Supplementary file 2 — Authors’ original file for figure 2 [file 12879_2014_653_MOESM2_ESM.gif]

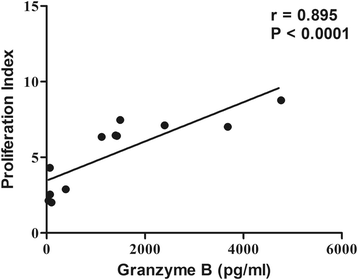

Supplement: Supplementary file 3 — Authors’ original file for figure 3 [file 12879_2014_653_MOESM3_ESM.gif]

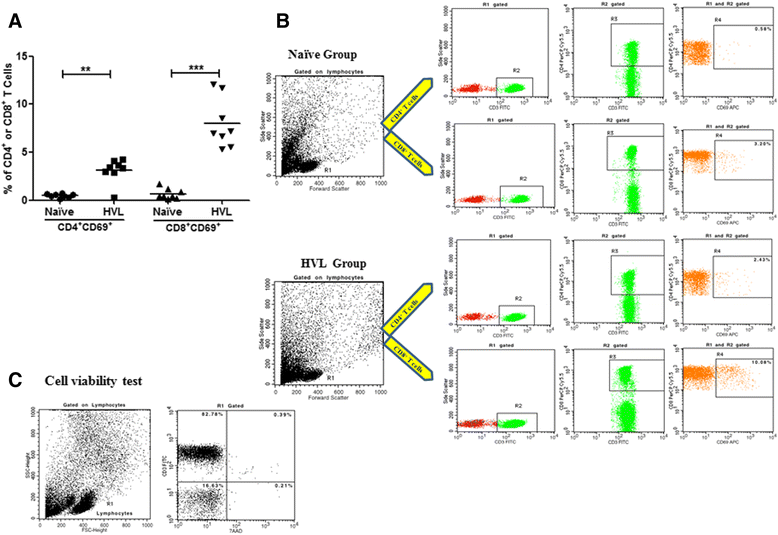

Supplement: Supplementary file 4 — Authors’ original file for figure 4 [file 12879_2014_653_MOESM4_ESM.gif]
